# Treatment and progression of patients in Sweden with metastatic castration-sensitive prostate cancer

**DOI:** 10.2340/1651-226X.2026.45110

**Published:** 2026-05-21

**Authors:** Johan Styrke, Åsa Jellvert, Marie Hjälm Eriksson, Fredrik Sandin, Elin Axén, Sam Jabbar, Kamilla Juhl Norgaard

**Affiliations:** a Department of Diagnostics and Intervention, Urology and Andrology, Umeå University, Umeå, Sweden; b Department of Oncology, Institute of Clinical Sciences, Sahlgrenska Academy, University of Gothenburg, Gothenburg, Sweden; c Department of Surgery, Region Västra Götaland, NU Hospital Group, Uddevalla, Sweden; d Department of Surgery and Oncology, Capio St. Görans Hospital, Stockholm, Sweden; e Department of Pathology and Oncology, Karolinska Institute, Stockholm, Sweden; f Regional Cancer Centre, Mid Sweden, Uppsala, Sweden; g Department of Urology, Institute of Clinical Sciences, Sahlgrenska Academy, University of Gothenburg, Gothenburg, Sweden; h Department of Urology, Sahlgrenska University Hospital, Region Västra Götaland, Gothenburg, Sweden; i Astellas Pharma A/S, Copenhagen, Denmark

**Keywords:** prostatic neoplasms, castration-resistant, Sweden, retrospective studies, disease progression

## Abstract

**Background and purpose:**

The metastatic castration-sensitive prostate cancer (mCSPC) treatment landscape in Sweden has evolved in recent years. The purpose of this study is to provide contemporary registry data on outcomes and treatment patterns in a nationwide cohort of Swedish patients with de novo mCSPC.

**Patient/material and methods:**

This retrospective cohort study included patients in Sweden with a clinical diagnosis of de novo mCSPC registered between January 1, 2017 and December 31, 2023. The primary outcome was time from de novo mCSPC diagnosis to mCRPC or death (2017–2023). Secondary outcomes included overall survival (OS), comorbidities, co-medication use, and treatment sequencing. OS, time to progression or death, and treatment sequencing for the 2017–2020 and 2021–2023 time periods were also analyzed.

**Results:**

A total of 2421 patients with de novo mCSPC were included in the primary analysis (mean age: 72.8 years). The median time to progression or death was 22.7 months (95% CI: 21.2–24.1), and the median OS was 49.8 months (95% CI: 47.0–52.6). Median time to progression or death increased from 19.9 months (95% CI: 18.6–21.2) in 2017–2020 to 30.0 months (95% CI: 26.6–33.3) in 2021–2023, while the proportion of patients receiving initial doublet or triplet therapy rose from 42.5 to 68.8%. No differences in OS were observed between different time periods.

**Interpretation:**

In Sweden (2017–2023), increased time to progression or death was observed in patients with de novo mCSPC.

## Introduction

Prostate cancer (PC) is the most common incident cancer among men worldwide, with 1.4 million cases occurring in 2023 [1]. Approximately 26,000 Nordic patients are diagnosed each year, and in 2022, around 310,000 were living with PC, with 5500 dying annually from the disease [2]. In Sweden, 63,957 patients were diagnosed with PC between 2019 and 2024, including 9942 patients in 2024, of whom 891 (9%) had distant metastatic PC [3]. Diagnosis rates, particularly for those with distant metastatic disease, remained relatively stable during this period [3].

**Figure 1:**
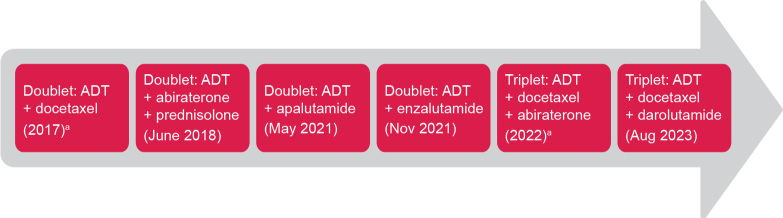
Introduction of new treatment options for mCSPC in Sweden based on Swedish Dental and Pharmaceutical Benefits Agency reimbursement dates. ADT: androgen-deprivation therapy. aNot reimbursed but included in Regional Cancer Center treatment guidelines.

Since 2017, several new treatments have been approved and reimbursed in Sweden for patients with metastatic castration-sensitive prostate cancer (mCSPC) (Figure 1), including doublet therapy (androgen-deprivation therapy [ADT] plus docetaxel or an androgen receptor pathway inhibitor [ARPI]) and triplet therapy (ADT plus docetaxel and an ARPI). However, few studies have investigated treatment patterns or outcomes in mCSPC after the approval of new treatment options [4–6], particularly in Sweden [7, 8]. The aim of this study was to provide contemporary registry data on outcomes and treatment patterns for patients with de novo mCSPC after the introduction of doublet and triplet therapy options in Sweden. We used the Prostate Cancer data Base Sweden (PCBase) [9] and Individual Patient Overview Prostate Cancer (Individuell Patientöversikt, IPÖ) to characterize the time to disease progression or death and overall survival (OS) for patients with de novo mCSPC (2017–2023). Additionally, we assessed comorbidities, co-medication use, and treatment sequencing for the 2017–2023 time period and evaluated time to progression or death, OS, and treatment sequencing between 2017–2020 and 2021–2023.

## Patients/material and methods

### Study design

This study was a retrospective observational incident cohort of patients in Sweden with clinically diagnosed de novo mCSPC (i.e. patients with metastatic disease at diagnosis, excluding those who developed mCSPC after an initial diagnosis of localized disease). This study was approved by the Swedish Research Ethics Authority, which waived the requirement for informed consent.

### Swedish cancer registers

The PCBase and IPÖ datasets were used to assess patients with de novo mCSPC. PCBase links data from the National Prostate Cancer Register (NPCR), the National Patient Register, the Swedish Prescribed Drug Register (SPDR), and the Cause of Death Register [7]. NPCR is a non-mandatory register that captures clinical and disease characteristics at diagnosis and first treatment, covering 98% of all patients diagnosed with PC in Sweden [7, 10]. The National Patient Register and SPDR contain diagnoses and treatments, respectively, associated with healthcare interactions [7, 10]. IPÖ is a follow-up tool for physicians to document disease progression and contains clinical information not reported in NPCR and other registers [7, 11]. Like NPCR, IPÖ is also a non-mandatory register, but it does not provide as high a coverage as NPCR, capturing approximately 36% of patients on androgen-receptor-targeting drugs [11].

### Inclusion and exclusion criteria

Data were included in the primary analysis set (i.e. ‘IPÖ cohort’) if the patient was registered in both PCBase and IPÖ and received a diagnosis of de novo mCSPC between January 1, 2017 and December 31, 2023. (Patients are initially registered in NPCR at the time of histological or clinical diagnosis of PC. Typically, radiological results are provided 3–4 weeks after diagnosis; if results suggest evidence of metastatic disease, patient diagnosis data are updated to specify de novo mCSPC.) In the sensitivity analysis set (i.e. ‘PCBase cohort’), all patients with mCSPC in PCBase were included regardless of registration in IPÖ. Patients who developed mCSPC after an initial diagnosis of localized disease were excluded from analysis, regardless of when progression to metastatic disease was noticed. Emigration dates from the registers were accounted for in this analysis.

### Endpoints

The primary outcome was the time from mCSPC diagnosis to mCRPC or all-cause death, whichever came first. Secondary outcomes included prevalence of co-medications most frequently prescribed in the year prior to de novo mCSPC diagnosis, prevalence of comorbidities (including Charlson Comorbidity Index [CCI] diagnosis groups) in the 10 years prior to de novo mCSPC diagnosis, and treatment sequencing. Time from de novo mCSPC diagnosis to progression or death, OS, and treatment sequencing for the 2017–2020 and 2021–2023 time periods were also analyzed. Information on date of death, as well as all follow up in IPÖ, were available up to February 24, 2024.

This analysis was conducted according to a predefined statistical plan. Descriptive statistics were used to summarize all collected variables of interest. All data processing, summarization, and analyses were performed using R Version 4.2.1 or higher. No imputation methods were applied to missing data.

Time-to-event endpoints were analyzed using the Kaplan–Meier (KM) product limit method. The cumulative incidence function per Kalbfleisch and Prentice [12] was used in the presence of competing risks to avoid overestimation and bias that can occur with the traditional KM method. The reverse KM method was applied to estimate median potential follow-up time; patients still alive at the end of follow-up when the final analysis was conducted were censored at that date.

Time from de novo mCSPC diagnosis to mCRPC or death (whichever came first) was estimated using the KM method (accounting for censoring), with median time presented as a 50th percentile estimate with a two-sided 95% CI. Progression to mCRPC was defined as the first occurrence in the IPÖ cohort of either a clinical assessment of mCRPC or any of the following signs of progression at least 3 months after de novo mCSPC diagnosis, assuming that all patients had ongoing treatment with ADT: (1) an increase of more than 1 ng/mL between two consecutive prostate-specific antigen (PSA) values above 2 ng/mL measured at least 1 week apart, accounting for the nadir; (2) new metastases or progression; or (3) treatment with cabazitaxel, carboplatin, carboplatin-etoposide, Radium-223 dichloride, cyclophosphamide, or docetaxel as a single agent after the end of doublet treatment with an ARPI.

Using data from SPDR, prevalence of co-medications most frequently prescribed in the year prior to de novo mCSPC diagnosis was assessed by Anatomical Therapeutic Chemical classification system codes according to the predefined statistical plan. Using data from the National Patient Register, comorbidities and CCI diagnosis groups in the 10 years prior to de novo mCSPC diagnosis were assessed according to International Classification of Disease Tenth Revision (ICD-10) codes as reported in Ludvigsson et al. [13]. Using data from IPÖ, NPCR, and SPDR, Sankey diagrams were generated to characterize treatment sequences in de novo mCSPC and to assess how recommended treatments were implemented. Patients undergoing doublet or triplet therapy were classified as such using the following scheme: doublet therapy was defined as the start of treatment with ADT plus docetaxel or an ARPI within 6 months of de novo mCSPC diagnosis and before progression to mCRPC; triplet therapy was defined as ADT plus docetaxel and an ARPI within 6 months of de novo mCSPC diagnosis.

An analysis of time to progression or death, OS, and treatment sequences was performed for the 2017–2020 and the 2021–2023 time periods.

## Results

### Number of participants and follow-up duration

**Figure 2:**
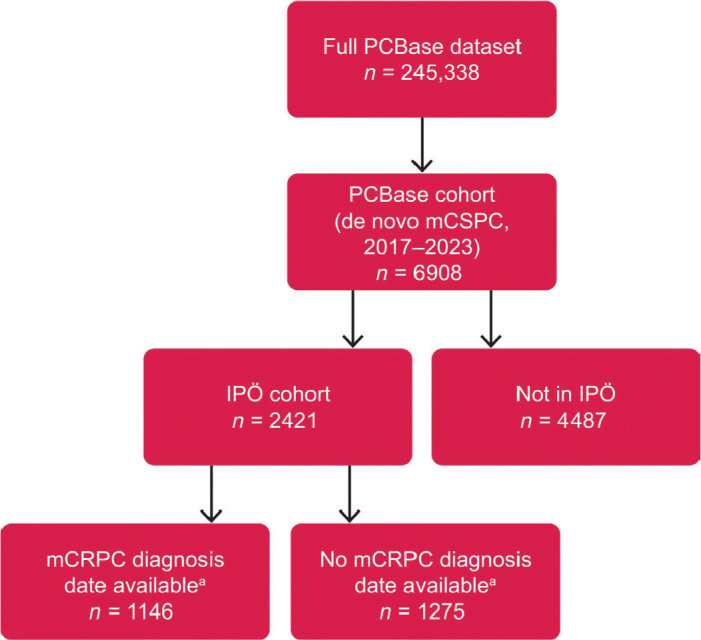
Data inclusion and analysis sets. mCRPC: metastatic castration-resistant prostate cancer; IPÖ: Individuell patientöversikt; PCBase: Prostate Cancer data Base Sweden. aThe date of progression to mCRPC was only available in IPÖ and recorded for approximately half of the IPÖ cohort until end of follow-up.

Of the 6908 patients with de novo mCSPC included in the PCBase cohort, 2421 were present in IPÖ and therefore constituted the IPÖ cohort (Figure 2). For patients in the IPÖ cohort, the median potential follow-up time from de novo mCSPC diagnosis was 43.2 months (95% CI: 41.3–46.9).

### Baseline characteristics, comorbidities, and co-medications

Baseline patient characteristics are shown in Table 1. The mean age of the IPÖ cohort was 72.8 years, the most frequent site of metastasis was bone (1227/1809; 67.8%), and approximately 50% of patients had one or more comorbidities listed in the CCI. Table S1 shows baseline characteristics of the IPÖ cohort by period (i.e. 2017–2020 and 2021–2023).

**Table 1:** Baseline characteristics of patients who received a clinical diagnosis of de novo mCSPC between January 1, 2017 and December 31, 2023.

	IPÖ Cohort *n*=2421		Not in IPÖ *n*=4487		PCBase Cohort *n*=6908	
**Age at diagnosis**						
Median (Min, Q1, Q3, Max)	73	40, 67, 78, 96	76	41, 70, 81, 100	75	40, 69, 80, 100
Mean (SD)	72.8	8.4	75.3	8.6	74.4	8.6
**Age at diagnosis**						
< 55	43	1.8	50	1.1	93	1.3
55–64	350	14.5	448	10.0	798	11.6
65–74	969	40.0	1471	32.8	2440	35.3
75–84	879	36.3	1888	42.1	2767	40.1
≥ 85	180	7.4	630	14.0	810	11.7
**PSA at diagnosis in ng/mL**						
< 50	779	32.2	1699	37.9	2478	35.9
50–99	322	13.3	589	13.1	911	13.2
100–499	740	30.6	1233	27.5	1973	28.6
≥ 500	568	23.5	916	20.4	1484	21.5
Missing	12	0.5	50	1.1	62	0.9
**Gleason score at diagnosis**						
6	11	0.5	48	1.1	59	0.9
7 (3+4)	87	3.6	231	5.1	318	4.6
7 (4+3)	263	10.9	513	11.4	776	11.2
8	437	18.1	758	16.9	1195	17.3
9–10	1428	59.0	2355	52.5	3783	54.8
Missing	195	8.1	582	13.0	777	11.2
**T-stage at diagnosis**						
T1	163	6.7	438	9.8	601	8.7
T2	581	24.0	1129	25.2	1710	24.8
T3	1192	49.2	1992	44.4	3184	46.1
T4	380	15.7	692	15.4	1072	15.5
TX	105	4.3	236	5.3	341	4.9
**N-stage at diagnosis**						
N0	727	30.0	1351	30.1	2078	30.1
N1	1004	41.5	1913	42.6	2917	42.2
NX	690	28.5	1223	27.3	1913	27.7
**Scintigraphy in assessment of skeletal metastases**						
Yes	1870	77.2	3338	74.4	5208	75.4
No	549	22.7	1145	25.5	1694	24.5
Missing	2	0.1	4	0.1	6	0.1
**CT in assessment of skeletal metastases**						
Yes	1012	41.8	1731	38.6	2743	39.7
No	1403	58.0	2751	61.3	4154	60.1
Missing	6	0.2	5	0.1	11	0.2
**PET/CT in assessment of skeletal metastases**						
Yes	307	12.7	515	11.5	822	11.9
No	2108	87.1	3968	88.4	6076	88.0
Missing	6	0.2	4	0.1	10	0.1
**MRI in assessment of skeletal metastases**						
Yes	297	12.3	560	12.5	857	12.4
No	2118	87.5	3922	87.4	6040	87.4
Missing	6	0.2	5	0.1	11	0.2
**Number of bone metastases**						
0	122	5.0	252	5.6	374	5.4
1–3	416	17.2	995	22.2	1411	20.4
≥ 4	811	33.5	1517	33.8	2328	33.7
Not assessed	1072	44.3	1723	38.4	2795	40.5
**Lung metastasis**						
Yes	175	7.2	399	8.9	574	8.3
No	2246	92.8	4088	91.1	6334	91.7
**Liver metastasis**						
Yes	63	2.6	197	4.4	260	3.8
No	2358	97.4	4290	95.6	6648	96.2
**Brain metastasis**						
Yes	5	0.2	24	0.5	29	0.4
No	2416	99.8	4463	99.5	6879	99.6
**Non-regional lymph node metastasis**						
Yes	234	9.7	391	8.7	625	9.0
No	2187	90.3	4096	91.3	6283	91.0
**Other metastasis**						
Yes	105	4.3	211	4.7	316	4.6
No	2316	95.7	4276	95.3	6592	95.4
**Radiotherapy within 6 months of diagnosis**						
Yes	199	8.2	368	8.2	567	8.2
No	2222	91.8	4119	91.8	6341	91.8
**ECOG at diagnosis**						
0	1112	45.9	0	0.0	1112	16.1
1	200	8.3	0	0.0	200	2.9
2+	122	5.0	0	0.0	122	1.8
Missing	987	40.8	4487	100.0	5474	79.2
**CCI**						
0	1344	55.5	2168	48.3	3512	50.8
1	697	28.8	1262	28.1	1959	28.4
2	245	10.1	615	13.7	860	12.4
3+	135	5.6	442	9.9	577	8.4
**Drug Comorbidity Index** ^**a**^						
Median (Min, Q1, Q3, Max)	0.9	−0.8, 0.2, 2.0, 12.1	1.3	−0.7, 0.4, 2.6, 14.8	1.1	−0.8, 0.3, 2.4, 14.8
Mean (SD)	1.3	1.5	1.7	1.7	1.6	1.7
**Multidimensional Diagnosis-based Comorbidity Index** ^**b**^						
Median (Min, Q1, Q3, Max)	0.1	−0.8, 0, 0.6, 4.8	0.3	−1.2, 0, 1.0, 5.1	0.2	−1.2, 0, 0.8, 5.1
Mean (SD)	0.4	0.7	0.6	0.8	0.5	0.8
**Estimated remaining lifetime**^**c**^ **(years)**						
Median (Min, Q1, Q3, Max)	12	1.2, 8.5, 16.2, 38.5	9.8	0.9, 6.2, 14.1, 34.7	10.7	0.9, 6.9, 15.0, 38.5
=Mean (SD)	12.6	5.8	10.7	5.8	11.4	5.9

Unless otherwise noted, numbers reported are n and %.

a Calculated from prescriptions within the last year before entry into mCSPC.

b Calculated from diagnoses within 10 years before entry into mCSPC.

c Calculated based on age, the Drug Comorbidity Index value, and the Multidimensional Diagnosis-based Comorbidity Index value. CCI: Charlson Comorbidity Index; ECOG: Eastern Cooperative Oncology Group; IPÖ: Individuell patientöversikt; Max: maximum; mCSPC: metastatic castration-sensitive prostate cancer; Min: minimum; MRI: Magnetic Resonance Imaging; PCBase: Prostate Cancer data Base Sweden; PSA: prostate-specific antigen; PET/CT: positron emission tomography/computed tomography; SD: standard deviation; Q1: first quartile; Q3: third quartile.

The IPÖ and PCBase cohorts, in general, had similar baseline characteristics, but those in the IPÖ cohort were slightly younger, had a slightly higher PSA and T-stage (T3) at diagnosis, and were less burdened with comorbidities as measured by both the Drug Comorbidity Index and the CCI (Table 1). No major differences were observed between the 2017–2020 and 2021–2023 time periods except the higher proportion of non-regional lymph nodes reported in 2021–2023 (13.1%) compared to 2017–2020 (7.0%) (Table S1). The most common comorbidities by ICD-10 code were hypertension (33.9%), age-related cataract (21.1%), atrial fibrillation and flutter (12.8%), and type 2 diabetes (11.6%) (Table 2A). The most common CCI diagnosis groups were malignancy (13.8%), cerebrovascular disease (10.0%), myocardial infarction (8.3%), and congestive heart failure (6.9%) (Table 2B). The most common co-medications included renin-angiotensin system agents (43.9%), antithrombotic agents (35.9%), analgesics (33.8%), and lipid modifying agents (33.7%) (Table 2C).

**Table 2:** Comorbidities and co-medications prior to mCSPC diagnosis, IPÖ cohort.

Code	Category	*n*	%
**A. Comorbidities by ICD-10 code 10 years prior to mCSPC diagnosis**			
I10	Essential (primary) hypertension	821	33.9
Z09	Encounter for follow-up examination after completed treatment for conditions other than malignant neoplasm	580	24.0
H25	Age-related cataract	511	21.1
Z96	Presence of other functional implants	353	14.6
I48	Atrial fibrillation and flutter	310	12.8
E11	Type 2 diabetes mellitus	282	11.6
E78	Disorders of lipoprotein metabolism and other lipidemias	281	11.6
I25	Chronic ischemic heart disease	267	11.0
R07	Pain in throat and chest	250	10.3
Z95	Presence of cardiac and vascular implants and grafts	241	10.0
M79	Other and unspecified soft tissue disorders, not elsewhere classified	237	9.8
Z03	Encounter for medical observation for suspected diseases and conditions ruled out	234	9.7
H35	Other retinal disorders	220	9.1
H40	Glaucoma	215	8.9
M54	Dorsalgia	200	8.3
**B. CCI diagnosis groups by ICD-10 code 10 years prior to mCSPC diagnosis**			
C00–C97, excluding C87	Malignancy	333	13.8
G45, I60–I64, I67, I69	Cerebrovascular disease	242	10.0
I21, I22, I25.2	Myocardial infarction	201	8.3
I11.0, I13.0, I13.2, I25.5, I42.0, I42.6–I42.9, I43, I50	Congestive heart failure	167	6.9
C77–C80	Metastatic solid tumor	144	5.9
M05, M06, M12.3, M07.0–M07.3, M13, M30, M31.3–M31.6, M32–M34, M35.0, M35.1, M35.3, M45, M46	Rheumatic disease	89	3.7
N03.2–N03.7, N05.2–N05.7, N11, N18, N19, N25.0, I12.0, I13.1, Q61.1–Q61.4, Z49, Z94.0, Z99.2	Renal disease	84	3.5
I70, I71, I73.1, I73.8, I73.9, I77.1, I79.0, I79.2 K55	Peripheral vascular disease	81	3.3
J43, J44	Chronic obstructive pulmonary disease	81	3.3
E10.2, E10.3, E10.4, E10.5, E10.7, E11.2–E11.7, E12.2–E12.7, E13.2–E13.7, E14.2–E14.7	Diabetes with chronic complication	80	3.3
J41, J42, J45–J47, J60–J70	Other chronic pulmonary disease	51	2.1
K25–K28	Peptic ulcer disease	33	1.4
G11.4, G80–G82, G83.0–G83.3, G83.8	Hemiplegia	29	1.2
F00–F03, F05.1, G30, G31.1, G31.9	Dementia	22	0.9
B15–B19, K73, K74.6, K70.3, K75.4	Mild liver disease	16	0.7
R18, I85.0, I85.9, I98.2, I98.3	Severe liver disease	10	0.4
E10.0, E10.1, E11.0–E11.1, E12.0–E12.1, E13.0–E13.1, E14.0–E14.1	Diabetes without chronic complication	2	0.1
B20–B24, F02.4, O98.7, R75, Z11.4, Z21.9, Z71.1	AIDS	0	0.0
**C. Co-medications by ATC code one year prior to mCSPC diagnosis**			
C09	Agents acting on the renin-angiotensin system	1062	43.9
B01	Antithrombotic agents	868	35.9
N02	Analgesics	819	33.8
C10	Lipid modifying agents	816	33.7
C07	Beta-blocking agents	717	29.6
J01	Antibacterials for systemic use	714	29.5
G04	Urologicals	655	27.1
C08	Calcium channel blockers	613	25.3
A02	Drugs for acid-related disorders	441	18.2
M01	Anti-inflammatory and antirheumatic products	437	18.1
C03	Diuretics	397	16.4
A06	Drugs for constipation	396	16.4
A10	Drugs used in diabetes	380	15.7
N05	Psycholeptics	342	14.1
B03	Antianemic preparations	337	13.9

ATC, Anatomic Therapeutic Chemical Classification System; mCSPC, metastatic castration-sensitive prostate cancer; ICD-10, International Classification of Diseases, 10th Revision; IPÖ, Individuell patientöversikt.

### Time from de novo mCSPC diagnosis to progression or death

**Figure 3:**
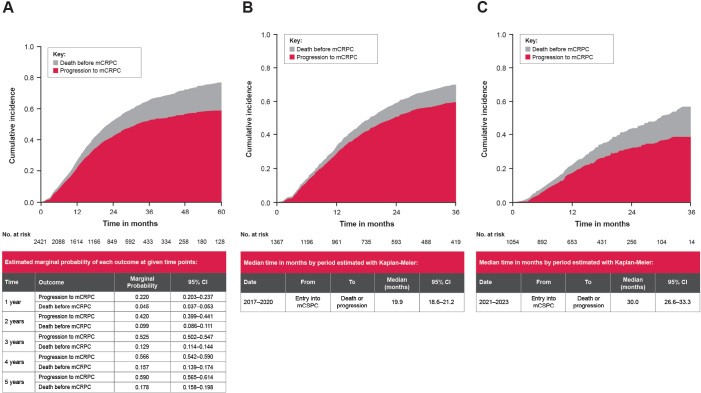
Cumulative incidence of mCSPC progression or death, IPÖ cohort. (A) de novo mCSPC diagnosis, 2017–2023. (B) de novo mCSPC diagnosis, 2017–2020. (C) de novo mCSPC diagnosis, 2021–2023. mCRPC: metastatic castration-resistant prostate cancer; mCSPC: metastatic castration-sensitive prostate cancer; IPÖ: Individuell patientöversikt

Patients with de novo mCSPC in the IPÖ cohort had a median time to progression or death of 22.7 months (95% CI: 21.2–24.1) (Figure 3A). At 3 years, risk of progression to mCRPC was 52.5% (95% CI: 50.2–54.7%), and risk of death before progression was 12.9% (95% CI: 11.4–14.4%). Median time to progression to mCRPC or death was 19.9 months (95% CI: 18.6–21.2) for patients diagnosed in 2017–2020 (Figure 3B) and 30.0 months (95% CI: 26.6–33.3) for those diagnosed in 2021–2023 (Figure 3C). Time from de novo mCSPC diagnosis to progression or death by age quartile is presented in Figure S1 (2017–2023) and Figure S2 (2017–2020 and 2021–2023).

**Figure 4:**
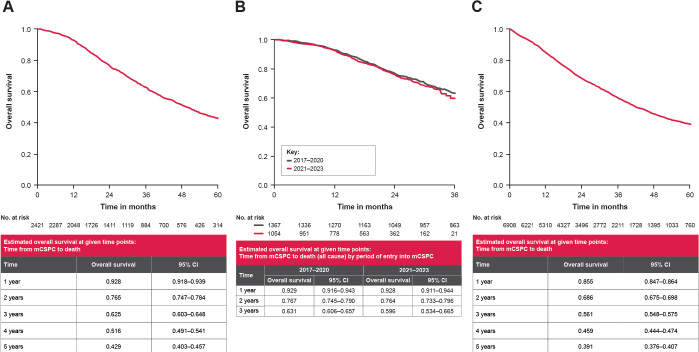
Kaplan–Meier graphs of time from mCSPC diagnosis to all-cause death. (A) de novo mCSPC diagnosis, 2017–2023, IPÖ cohort. (B) de novo mCSPC diagnosis, 2017–2020 and 2021–2023, IPÖ cohort. (C) de novo mCSPC diagnosis, 2017–2023, PCBase cohort. mCSPC: metastatic castration-sensitive prostate cancer; IPÖ: Individuell patientöversikt; PCBase: Prostate Cancer data Base Sweden.

OS rates and median follow-up times were assessed for patients in both the IPÖ and PCBase cohorts. For patients in the IPÖ cohort, the median OS was 49.8 months (95% CI: 47.0–52.6) (Figure 4A), with an OS rate at 3 years of 62.5% (95% CI: 60.3–64.8%). Analysis of OS showed similar outcomes between 2017–2020 and 2021–2023 (Figure 4B). For patients in the PCBase cohort, median survival was 42.4 months (95% CI: 40.8–44.7) (Figure 4C), with an OS rate at 3 years of 56.1% (95% CI: 54.8–57.5%). The cumulative incidence of mCSPC progression or death from PC or other causes in 2017–2022 is shown in Figure S3.

### Treatment sequencing

Of the 2421 total patients in the IPÖ cohort, 2321 are depicted in the Sankey diagrams: 94 lacked recorded treatments in IPÖ, and six received alternative treatments that are not represented in the Sankey diagrams.

**Figure 5:**
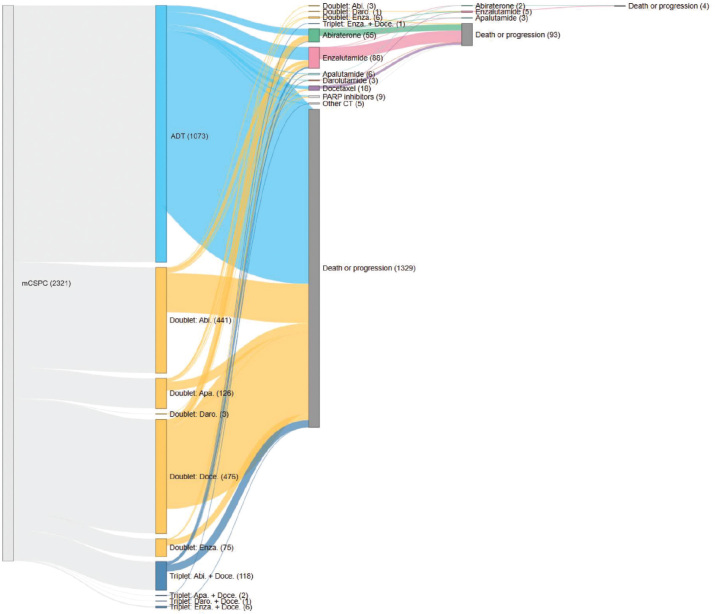
Sequence of treatments in de novo mCSPC, 2021–2023, IPÖ cohort. The count varies from the overall total of 2421 patients within the IPÖ dataset. Out of the 100 patients that are absent from this count, 94 lacked recorded treatments in IPÖ for the mCSPC phase. In addition, six patients received alternative treatments that are not represented in the Sankey diagram. ‘Death or progression’ means that the patient has either died or progressed to mCRPC and is therefore not receiving further treatments within the mCSPC phase. Abi: abiraterone; ADT: androgen-deprivation therapy; Apa: apalutamide; CT: chemotherapy; Daro: darolutamide; Doce: docetaxel; Enza: enzalutamide; IPÖ: Individuell patientöversikt; mCSPC: metastatic castration-sensitive prostate cancer; PARP: poly (ADP-ribose) polymerase; Ra223: radium‑223 dichloride.

Treatment sequencing from de novo mCSPC diagnosis to progression, death, or end of follow up is shown in Figure 5. Only a small proportion of patients were given additional treatment after initial doublet or triplet therapy. Overall, the most common treatment in mCSPC was ADT monotherapy (1073/2321; 46.2%), followed by various combinations of doublet therapies (1121/2321; 48.3%) (Figure 5). Among doublet therapies, the most common doublet was ADT plus docetaxel (476/1121; 42.5%), followed by ADT plus abiraterone with prednisolone (441/1121; 39.3%) (Figure 5). During 2017–2020, more than half of patients overall received ADT monotherapy as the first treatment for mCSPC (763/1328; 57.5%), followed by ADT plus docetaxel (367/1328; 27.6%) and ADT plus abiraterone with prednisolone (154/1328; 11.6%) (Figure S4). Triplet therapy accounted for 1.7% of treatments (22/1328) (Figure S4). However, during 2021–2023, only 31% of patients overall (310/993) received ADT monotherapy as the first treatment for mCSPC, followed by ADT plus abiraterone with prednisolone (287/993; 28.9%), ADT plus apalutamide (125/993; 12.6%), and ADT plus docetaxel (109/993; 11.0%) (Figure S5). Triplet therapy accounted for 10.6% of treatments (105/993) (Figure S5).

Full treatment sequencing from de novo mCSPC diagnosis to death or end of follow up is shown in Figure S6. Many patients (993/2321; 42.8%) received a second treatment, likely due to new indications or toxicity from the first treatment, after which they either received subsequent treatment or died (Figure S6).

## Discussion

This large retrospective Swedish registry analysis provides a comprehensive understanding of mCSPC treatment and progression in Sweden and shows an increased time from diagnosis to disease progression or death during the 2017–2023 time period. Our population had a high rate of age-related comorbidities, including hypertension, cardiovascular diseases, diabetes, and hyperlipidemia; co-medication use was consistent with this observation.

In the context of an evolving treatment landscape, a longer median OS was observed for this nationwide cohort of patients in Sweden with de novo mCSPC (2017–2023) compared to previous cohorts derived from PCBase over the past decade [8, 14]. In a study by Westerberg et al., median OS for patients in Sweden with de novo mCSPC increased from 2.2 years (1998–2001) to 2.7 years (2010–2015); this was likely explained by earlier detection as well as use of chemotherapy in CRPC [14]. According to Corsini et al., the restricted mean survival increased from 2.7 years (2008–2012) to 3.2 years (2017–2020), likely due to introduction of doublet therapy [8]; a further extension in survival was observed in our PCBase cohort (2017–2023), where median OS approximated 42 months (3.5 years). Compared to the PCBase cohort, patients in the IPÖ cohort had a slightly higher OS of approximately 50 months (4.2 years), likely explained by their baseline characteristics: IPÖ patients were slightly younger and healthier but had higher Gleason scores, PSA levels, and T-stages at diagnosis, suggesting that a higher treatment intensity, in combination with a higher life expectancy, could explain the difference in OS.

In Sweden, adoption of doublet therapy began in 2017 [15], following publication of CHAARTED [16] and STAMPEDE [17] trial results, which demonstrated improved OS when docetaxel was added to castration therapy for newly diagnosed metastatic PC. With the docetaxel patent expiration, its use increased significantly [8]. Subsequently, two additional studies established efficacy of ADT plus abiraterone with prednisolone as a doublet regimen [18, 19], which became the guideline-recommended standard in Sweden by 2018 [20]. However, uptake of this therapy for de novo mCSPC remained slow through 2020 due to local/regional variations in adoption [20].

In our study, doublet therapy with ADT plus abiraterone with prednisone increased from 11.6% of de novo mCSPC cases (2017–2020) to 28.9% of cases (2021–2023). Other doublet therapies used in the study included ADT plus apalutamide and ADT plus enzalutamide, both of which were reimbursed in Sweden by 2021 [21]. Docetaxel use in first-line doublet therapy rapidly decreased from 27.6% of de novo mCSPC cases in 2017–2020 to 11.0% of cases in 2021–2023. The likely explanation for this decrease is the abiraterone patent expiration in 2022 and the subsequent increase in triplet therapy use from 1.7% (2017–2020) to 10.6% (2021–2023). The PEACE-1 study by Fizazi et al. showed that triplet therapy with ADT plus docetaxel plus abiraterone with prednisolone increased survival in mCSPC [22], with Smith et al. showing similar results in an mCSPC population with the combination of ADT plus docetaxel plus darolutamide [23]. In our study, overall use of doublet or triplet therapy in mCSPC increased from 42.5 to 68.8% between 2017–2020 and 2021–2023, indicating that undertreatment is likely decreasing in Sweden.

The median (min, max) ages in our PCBase cohort were 75 (40, 100) years and 73 (40, 96) years in the IPÖ cohort. In general, the expected remaining lifetime is very important in clinical decision-making for oncological treatments, taking age and comorbidities into account. In terms of ARPI treatments in the elderly population, subgroup analyses have shown promising results across studies [18, 24–26]. While studies of enzalutamide and of abiraterone with prednisolone in mCSPC did not find a statistically significant improvement in OS for the subgroup of elderly patients aged 75 years and older, trends were positive [18, 19, 27]. Two studies on ARPI treatment in mCSPC reported significantly prolonged OS in the elderly subgroup, specifically for enzalutamide (patients ≥ 70 years of age) and apalutamide (patients ≥ 75 years of age) [25, 26]. Similar findings were observed with docetaxel. The CHAARTED study showed a significant increase in OS following docetaxel treatment in patients ≥ 70 years of age [16], while the STAMPEDE study did not show this benefit for patients in the same age group [17]. It should be noted that these findings were based on subgroup analyses in studies that were not designed to focus on age. Overall, however, the findings suggest that elderly patients may be more challenging to treat, given that OS in our analysis did not improve in 2021–2023 as more patients received doublet or triplet therapy.

Within 5 years of diagnosis, cumulative incidence of progression to mCRPC was 59.0%, and cumulative incidence of death before progression was 17.8%. Cumulative incidence of deaths before progression was higher in 2021–2023 than in 2017–2020 (18.2 vs. 11.3% at 3 years). The early and late IPÖ cohorts were comparable in age, PC staging, and life expectancy, suggesting that baseline characteristics cannot explain the variation. A possible explanation is that doublet therapy, which is commonly used, may keep patients in the mCSPC phase for a longer time, leading to a higher risk of death from other causes before progression to mCRPC. Another possible explanation is that, compared to ADT monotherapy, doublet and triplet therapy could lead to higher complication rates and deaths from side effects, such as cardiovascular disease.

Data indicate that patients diagnosed in 2021–2023 experienced a prolonged mCSPC phase compared with those diagnosed in 2017–2020, including patients in the oldest age quartile. This likely reflects increased treatment intensification in the mCSPC setting during the 2021–2023 period. Despite the prolonged mCSPC phase, OS did not improve between the two periods, and a numerical decrease in OS was observed in the oldest patient group. The reasons for this finding remain uncertain. One possible explanation is that an extended mCSPC phase is followed by a shortened mCRPC phase, as elderly and frail patients may be unable to receive further life-prolonging treatments after progression. From a patients’ perspective, prolonged time in the mCSPC phase may nevertheless be clinically meaningful, given the lower symptom burden compared with mCRPC. The potential decrease in OS among the oldest patients, however, raises concerns. If confirmed, it may suggest that intensified combination therapy in mCSPC confers limited benefit or even harm in selected elderly patients, possibly due to treatment-related toxicity or increased frailty. Although the present study cannot address cause-specific mortality, these findings warrant further investigation in updated analyses of NPCR data and other population-based PC registries.

Certain conditions are of particular concern with doublet or triplet therapy. Chronic heart failure, uncontrolled ischemic heart disease, and hypertension warrant caution, as ARPIs have been associated with different cardiac adverse events [28–30]. Each ARPI has a distinct cardiovascular event profile [29], and the European Society of Cardiology guidelines for cardio-oncology recommend selecting PC treatments based on pre-existing comorbidities, as well as monitoring for cardiovascular issues in patients receiving these therapies [31]. In this study, chronic ischemic heart disease was identified in 11% of patients and hypertension in 34% (IPÖ cohort). Future research should investigate how well these patients tolerate doublet or triplet therapy to further optimize individualized treatment decisions.

This analysis had various strengths and limitations. Firstly, the focus on de novo mCSPC offers a unique opportunity to compare study populations identified by NPCR and IPÖ. IPÖ is the only Swedish register that documents PC disease progression based on laboratory tests, radiologic progression, and clinician judgment; it also records chemotherapy treatments, which are not included in SPDR. However, registration in IPÖ is voluntary and only captures a subset of PC patients. Patients in the IPÖ cohort were younger and had a higher PSA level and Gleason score compared with the PCBase cohort, reflecting a possible selection toward those who are fit for multiple treatments. Secondly, patients with de novo mCSPC identified in 2021–2023 had shorter follow-up periods for assessing OS than those identified in 2017–2020. Thus, the finding of no difference in OS may be partially explained by the shorter follow-up time for patients with de novo mCSPC identified between 2021–2023 versus those identified from 2017–2020. Thirdly, causes of death other than PC were not accounted for in this analysis. Future studies should assess whether intensified treatment in mCSPC leads to causes of death other than PC. Fourthly, the IPÖ database presently contains minimal quality of life (QoL) data. While it is possible to register and monitor QoL data in IPÖ, the present coverage is low, making any data interpretation difficult. Future analyses should consider comparing QoL in mCSPC versus mCRPC in the light of the prolonged mCSPC phase shown in this study. Lastly, in more than 50% of cases, the number of bone metastases was missing; however, this did not affect primary outcome measurements.

## Conclusion

In the context of an evolving treatment landscape in Sweden, increased time to progression or death – likely resulting from higher treatment intensity – was observed in patients with de novo mCSPC (2017–2023). OS did not improve during this period, and a slight decrease in OS was observed in the oldest quartile, warranting further studies on treatment effects in elderly patients. This study emphasizes the importance of nationwide registry data to monitor the post-authorization effects of therapies on disease progression.

## Supplementary Material



## Data Availability

Data available: No. Explanation for why data are not available: All data generated or analyzed during this study, which support the findings of this study, are included within this article and its supplementary information files. Researchers interested in further analysis not present in the manuscript may contact the corresponding author.
