# Supplementary material for: Treatment and progression of patients in Sweden with metastatic castration-sensitive prostate cancer
Source: Acta Oncol. 2026 May 21;65:45110. doi: 10.2340/1651-226X.2026.45110 (PMC13200255; doi:10.2340/1651-226X.2026.45110)
Supplement: Supplementary file 1 [file AO-65-45110-s1.pdf]

Supplementary material has been published as submitted. It has not been copyedited, or typeset by Acta Oncologica

Supplemental tables/illustrations/figures

Table S1. *Baseline characteristics of patients in the IPÖ cohort who received a clinical diagnosis of de novo mCSPC between January 1, 2017 and December 31, 2023, by period*

Characteristic	Patients, No. (%)			
	2017–2020		2021–2023	
**No. of patients**	**1367**		**1054**	
**Age at diagnosis**				
Median (Min, Q1, Q3, Max)	73	(48, 68, 78, 96)	74	(40, 67, 79, 95)
Mean (SD)	72.8	(8.2)	72.9	(8.6)
**Age at diagnosis**				
<55	20	(1.5)	23	(2.2)
55–64	200	(14.6)	150	(14.2)
65–74	579	(42.4)	390	(37.0)
75–84	467	(34.2)	412	(39.1)
≥85	101	(7.4)	79	(7.5)
**PSA at diagnosis in ng/mL**				
<50	420	(30.7)	359	(34.1)
50–99	167	(12.2)	155	(14.7)
100–499	439	(32.1)	301	(28.6)
≥500	333	(24.4)	235	(22.3)
Missing	8	(0.6)	4	(0.4)
**Gleason score at diagnosis**				
6	6	(0.4)	5	(0.5)
7 (3+4)	49	(3.6)	38	(3.6)
7 (4+3)	135	(9.9)	128	(12.1)
8	252	(18.4)	185	(17.6)
9–10	814	(59.5)	614	(58.3)
Missing	111	(8.1)	84	(8.0)
**T-stage at diagnosis**				

T1	99	(7.2)	64	(6.1)
T2	338	(24.7)	243	(23.1)
T3	661	(48.4)	531	(50.4)
T4	222	(16.2)	158	(15.0)
TX	47	(3.4)	58	(5.5)
**N-stage at diagnosis**				
N0	400	(29.3)	327	(31.0)
N1	533	(39.0)	471	(44.7)
NX	434	(31.7)	256	(24.3)
**Scintigraphy in assessment of skeletal metastases**				
Yes	1083	(79.2)	787	(74.7)
No	282	(20.6)	267	(25.3)
Missing	2	(0.1)	0	(0.0)
**CT in assessment of skeletal metastases**				
Yes	557	(40.7)	455	(43.2)
No	804	(58.8)	599	(56.8)
Missing	6	(0.4)	0	(0.0)
**PET/CT in assessment of skeletal metastases**				
Yes	141	(10.3)	166	(15.7)
No	1220	(89.2)	888	(84.3)
Missing	6	(0.4)	0	(0.0)
**MRI in assessment of skeletal metastases**				
Yes	197	(14.4)	100	(9.5)
No	1164	(85.1)	954	(90.5)
Missing	6	(0.4)	0	(0.0)
**Number of bone metastases**				
0 metastases	60	(4.4)	62	(5.9)
1–3 metastases	188	(13.8)	228	(21.6)
4 or more metastases	403	(29.5)	408	(38.7)
Not assessed	716	(52.4)	356	(33.8)
**Lung metastasis**				
Yes	101	(7.4)	74	(7.0)

No	1266	(92.6)	980	(93.0)
**Liver metastasis**				
Yes	43	(3.1)	20	(1.9)
No	1324	(96.9)	1034	(98.1)
**Brain metastasis**				
Yes	4	(0.3)	1	(0.1)
No	1363	(99.7)	1053	(99.9)
**Non-regional lymph node metastasis**				
Yes	96	(7.0)	138	(13.1)
No	1271	(93.0)	916	(86.9)
**Other metastasis**				
Yes	65	(4.8)	40	(3.8)
No	1302	(95.2)	1014	(96.2)
**Radiotherapy within 6 months of diagnosis**				
Yes	64	(4.7)	135	(12.8)
No	1303	(95.3)	919	(87.2)
**ECOG at diagnosis**				
0	795	(58.2)	317	(30.1)
1	62	(4.5)	138	(13.1)
2+	38	(2.8)	84	(8.0)
Missing	472	(34.5)	515	(48.9)
**CCI**				
0	749	(54.8)	595	(56.5)
1	401	(29.3)	296	(28.1)
2	148	(10.8)	97	(9.2)
3+	69	(5.0)	66	(6.3)
**Drug Comorbidity Index**[a]				
Median (Min, Q1, Q3, Max)	0.9	(−0.7, 0.2, 2.0, 10.2)	0.8	(−0.8, 0.1, 1.9, 12.1)
Mean (SD)	1.4	(1.6)	1.3	(1.5)
**Multidimensional Diagnosis-based Comorbidity Index**[b]				
Median (Min, Q1, Q3, Max)	0.1	(−0.8, 0, 0.6, 4)	0.1	(−0.8, 0, 0.6, 4.8)
Mean (SD)	0.4	(0.7)	0.4	(0.7)

**Estimated Remaining Lifetime[c] (Years)**				
Median (Min, Q1, Q3, Max)	12.2	(1.2, 8.6, 16.2, 32.1)	11.9	(1.4, 8.2, 16.1, 38.5)
Mean (SD)	12.7	(5.6)	12.6	(6.0)

[a] Calculated from prescriptions within the last year before entry into mCSPC.

[b] Calculated from diagnoses within 10 years before entry into mCSPC.

[c] Calculated based on age, the Drug Comorbidity Index value, and the Multidimensional Diagnosis-based Comorbidity Index value.

CCI, Charlson Comorbidity Index; ECOG, Eastern Cooperative Oncology Group; IPÖ, Individuell patientöversikt; Max, maximum; mCSPC, metastatic castration-sensitive prostate cancer; Min, minimum; PCBase, Prostate Cancer data Base Sweden; PSA, prostate-specific antigen; PET/CT, positron emission tomography/computed tomography; Q1, first quartile; Q3, third quartile.

Figure S1. *Time from de novo mCSPC diagnosis to death by age quartile, 2017–2020 and 2021–2023, IPÖ cohort*

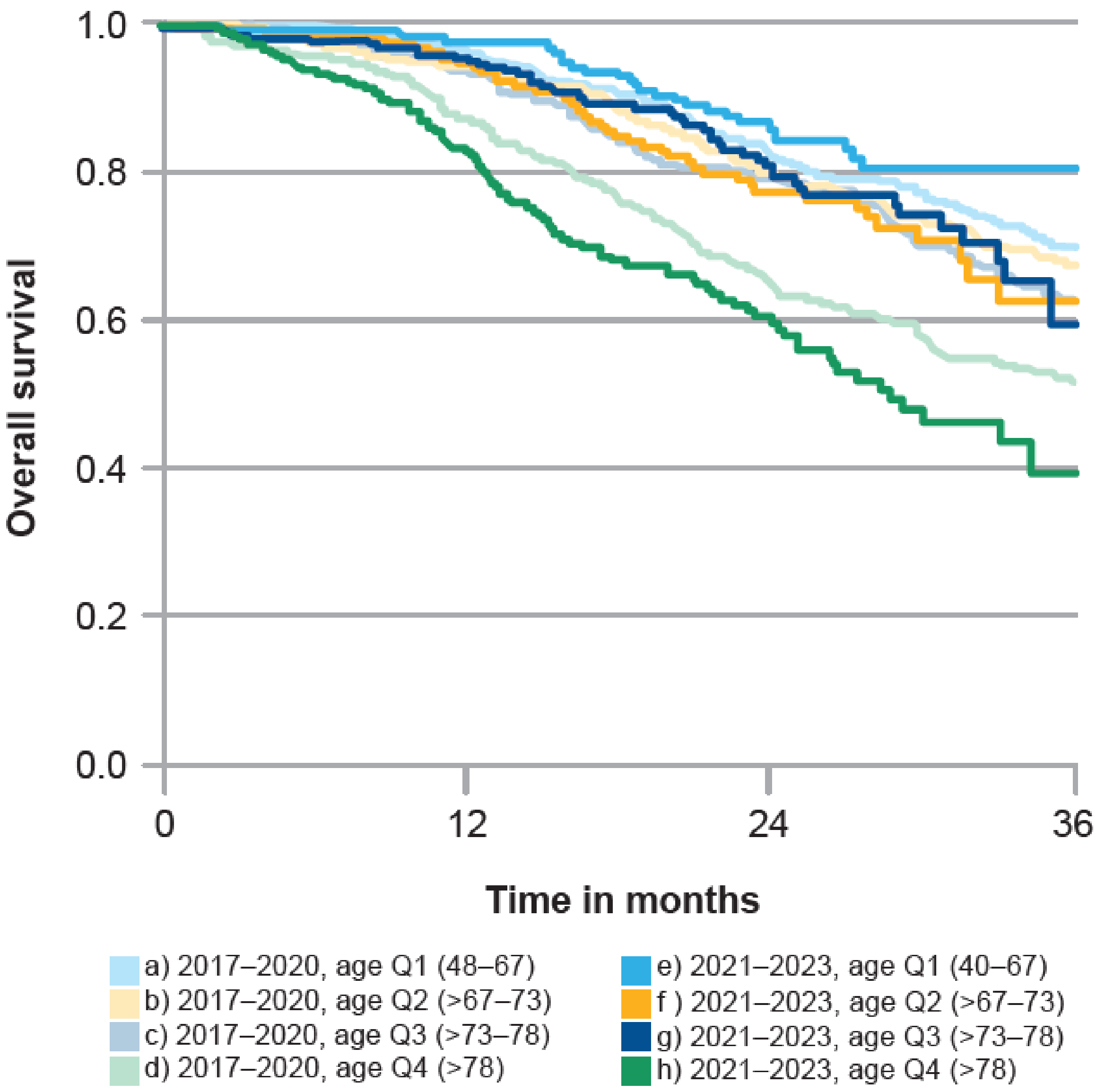

No. at risk

a)	338	337	326	305	278	261	236
b)	385	376	362	340	307	284	260
c)	313	306	293	265	248	220	195
d)	331	317	289	253	216	192	172
e)	268	247	207	149	102	48	4
f)	245	223	181	134	84	37	8
g)	270	242	212	166	103	49	7
h)	271	239	178	114	73	28	2

Figure S2. *Time from de novo mCSPC diagnosis to death or progression by age quartile, 2017–2020 and 2021–2023, IPÖ cohort*

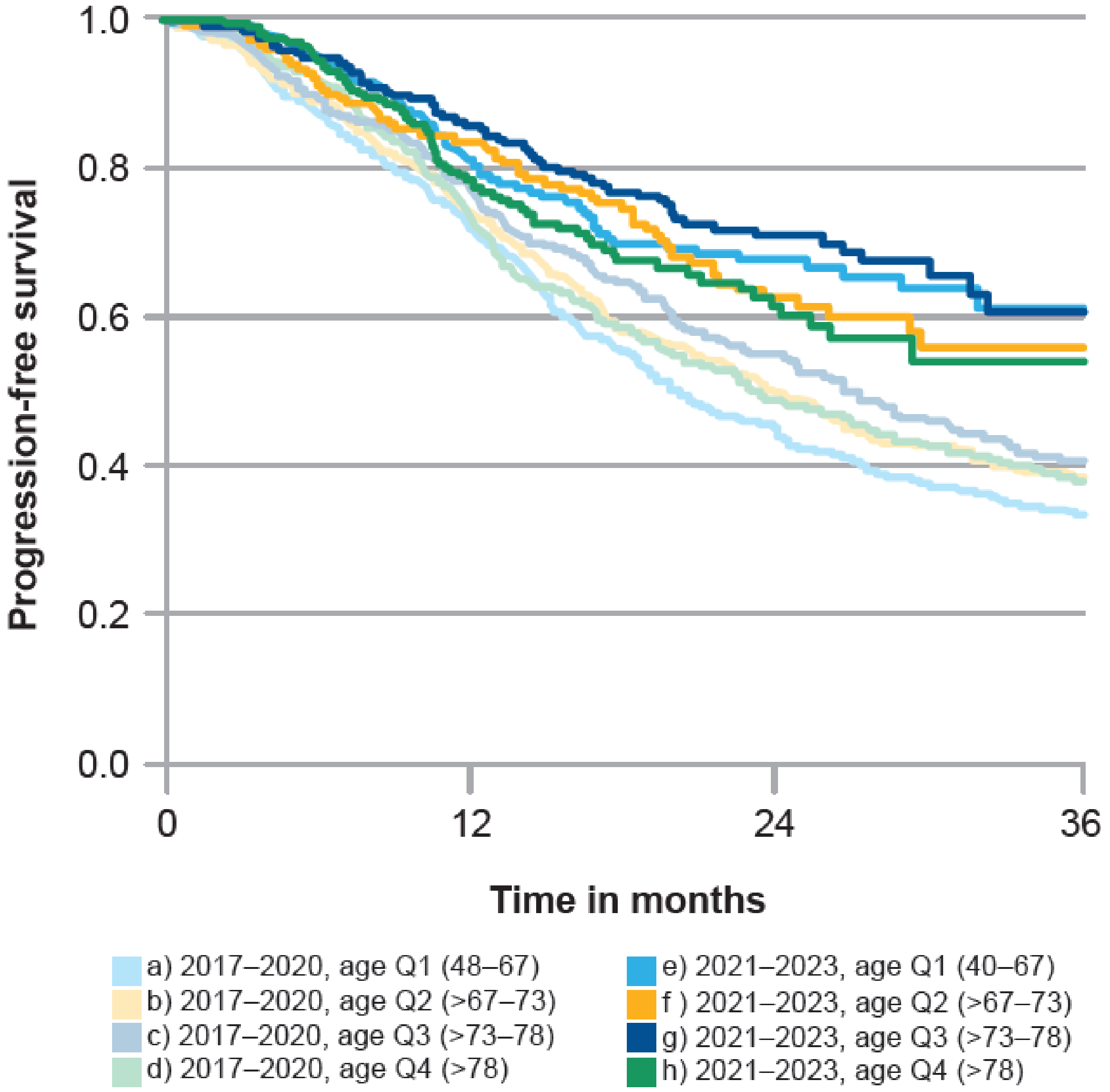

No. at risk

a)	338	294	239	180	143	117	102
b)	385	338	272	207	169	141	123
c)	313	273	229	184	154	125	106
d)	331	291	221	164	127	105	88
e)	268	234	168	108	73	31	1
f)	245	203	157	109	55	21	6
g)	270	230	183	131	77	36	5
h)	271	225	145	83	51	16	2

Figure S3. *Cumulative incidence of mCSPC progression or death from prostate cancer or other causes, 2017–2022, IPÖ cohort*

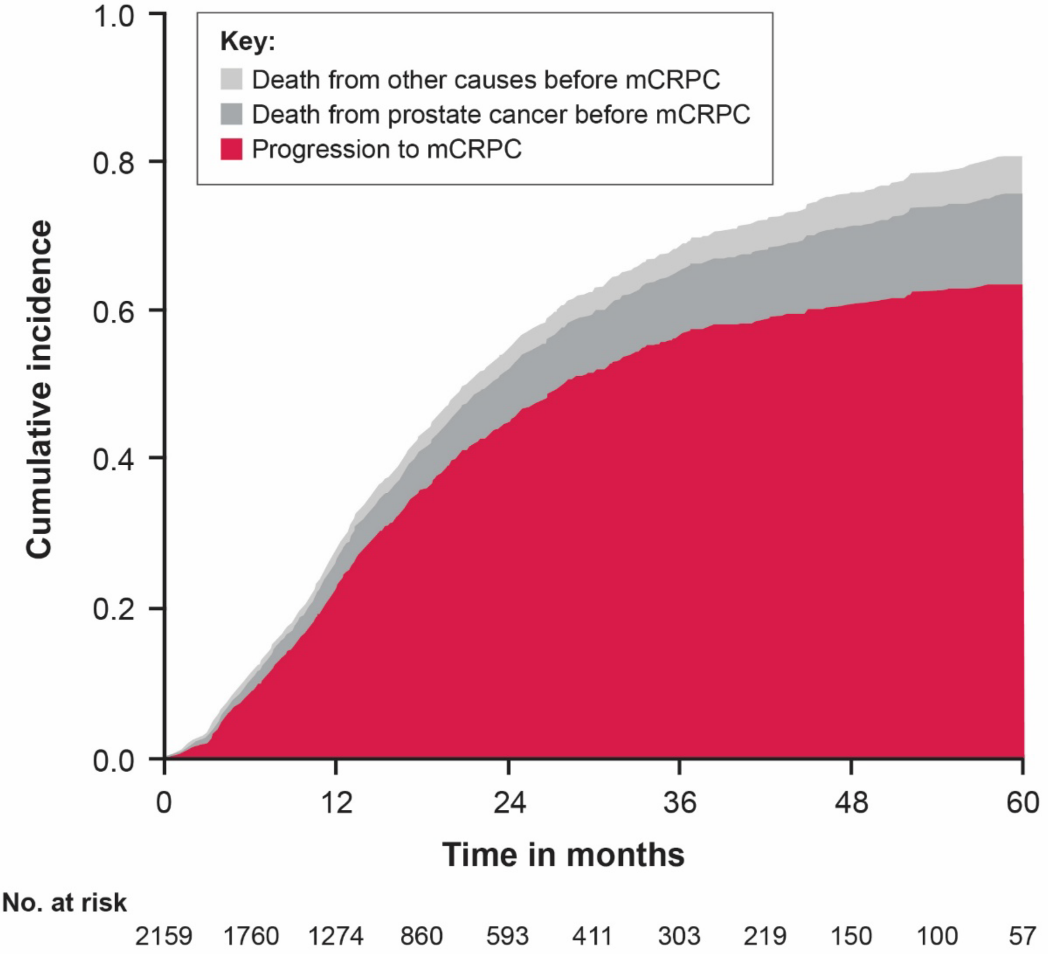

Estimated marginal probability of each outcome at given time points:			
**Time**	**Outcome**	**Marginal Probability**	**95% CI**
1 year	Progression to mCRPC	0.233	0.214–0.252
	Death from prostate cancer before mCRPC	0.033	0.025–0.041
	Death from other causes before mCRPC	0.013	0.008–0.018
2 years	Progression to mCRPC	0.453	0.429–0.477
	Death from prostate cancer before mCRPC	0.068	0.056–0.080
	Death from other causes before mCRPC	0.027	0.020–0.035
3 years	Progression to mCRPC	0.571	0.545–0.597
	Death from prostate cancer before mCRPC	0.084	0.071–0.098
	Death from other causes before mCRPC	0.034	0.025–0.043
4 years	Progression to mCRPC	0.612	0.585–0.638
	Death from prostate cancer before mCRPC	0.104	0.087–0.121
	Death from other causes before mCRPC	0.045	0.033–0.056
5 years	Progression to mCRPC	0.638	0.610–0.667
	Death from prostate cancer before mCRPC	0.120	0.100–0.140
	Death from other causes before mCRPC	0.052	0.038–0.065

Figure S4. *Sequence of treatments in de novo mCSPC, 2017–2020, IPÖ cohort*[a]

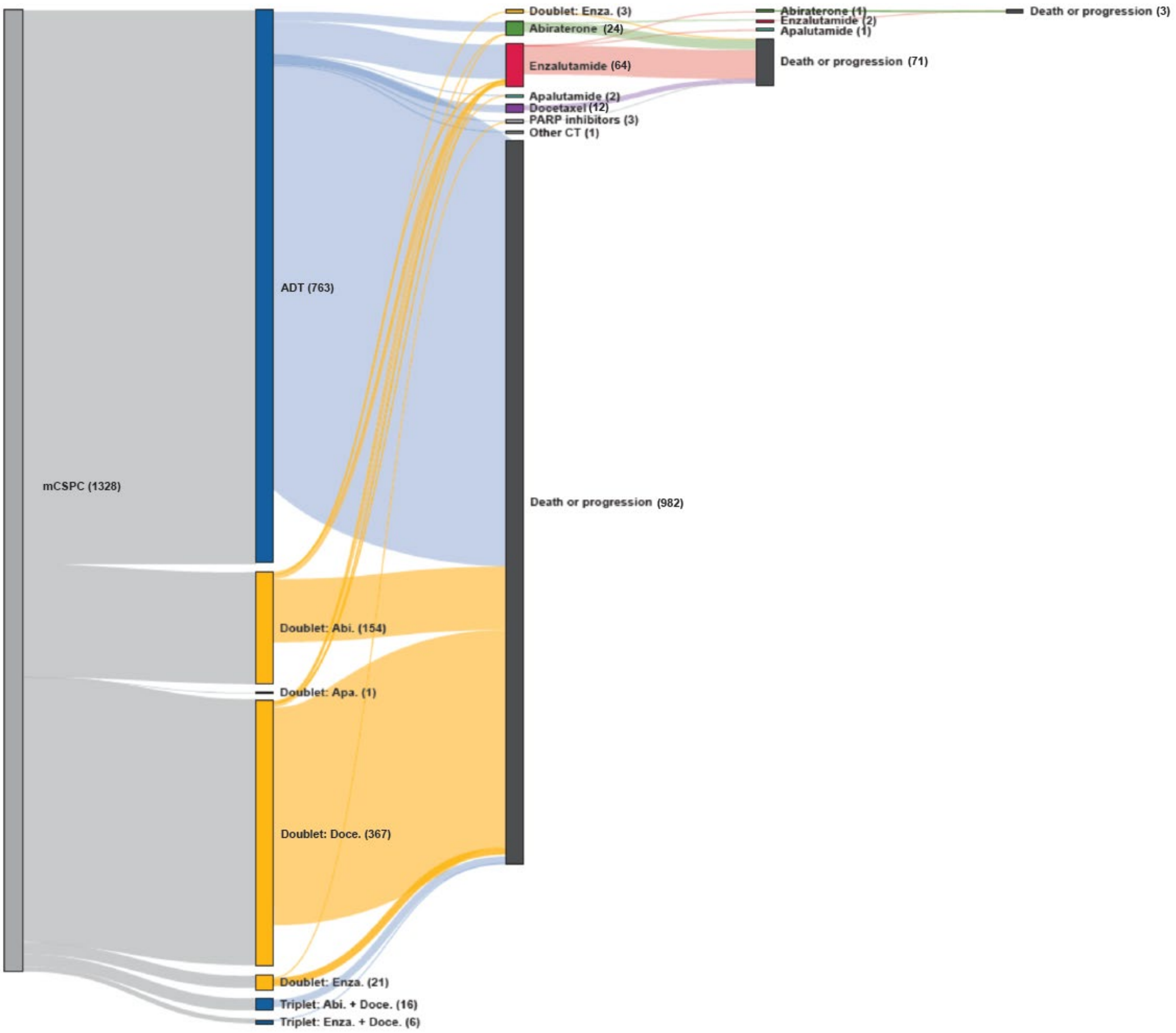

Abi, abiraterone; ADT, androgen-deprivation therapy; Apa, apalutamide; CT, chemotherapy; Daro, darolutamide; Doce, docetaxel; Enza, enzalutamide; IPÖ, Individuell patientöversikt; mCSPC, metastatic castration-sensitive prostate cancer; PARP, poly (ADP-ribose) polymerase; Ra223, radium-223 dichloride.

[a] "Death or progression" means that the patient has either died or progressed to mCRPC and is therefore not receiving further treatments within the mCSPC phase.

Figure S5. *Sequence of treatments in de novo mCSPC, 2021–2023,* IPÖ cohort[a]

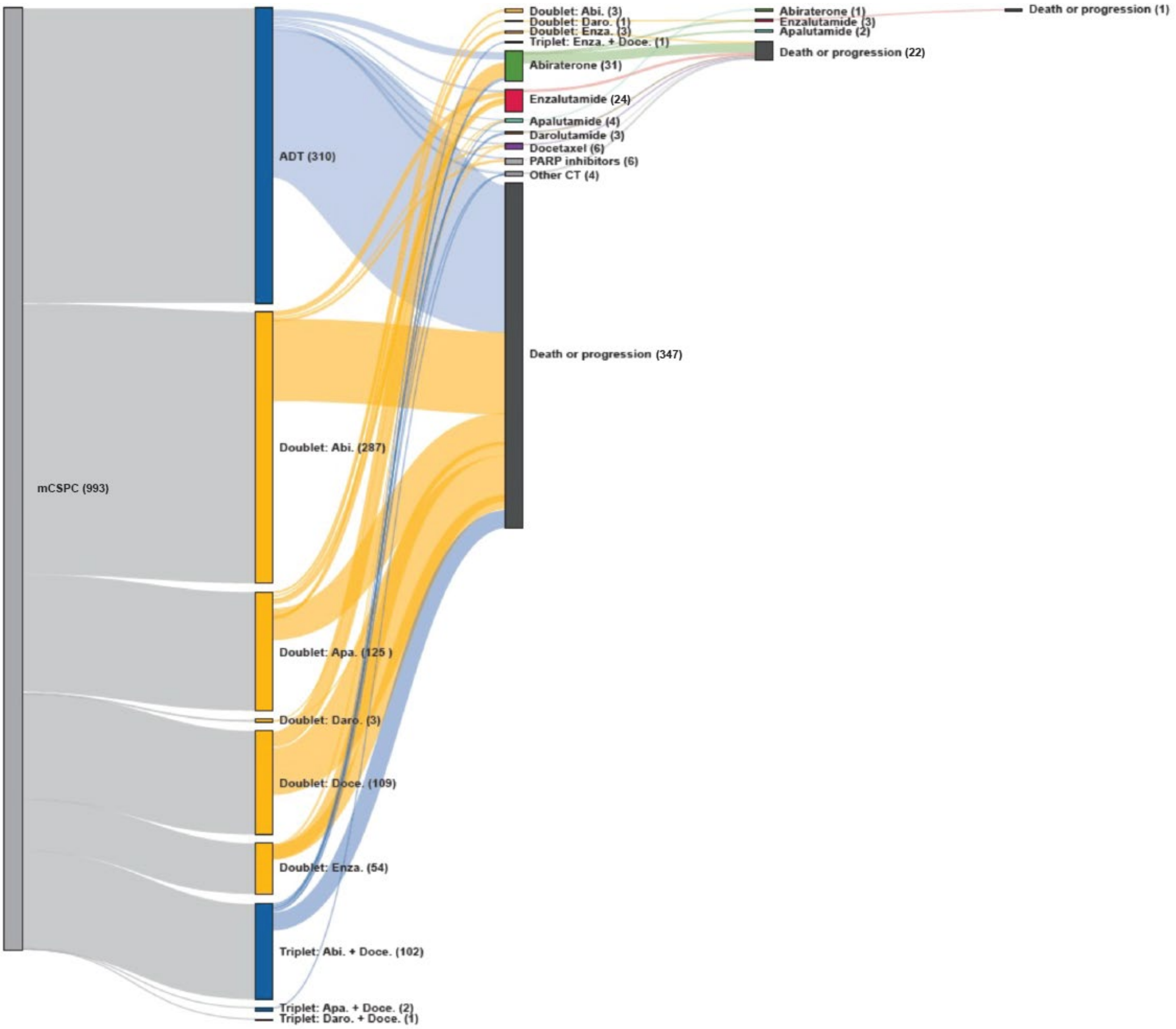

Abi, abiraterone; ADT, androgen-deprivation therapy; Apa, apalutamide; CT, chemotherapy; Daro, darolutamide; Doce, docetaxel; Enza, enzalutamide; IPÖ, Individuell patientöversikt; mCSPC, metastatic castration-sensitive prostate cancer; PARP, poly (ADP-ribose) polymerase; Ra223, radium-223 dichloride.

[a] "Death or progression" means that the patient has either died or progressed to mCRPC and is therefore not receiving further treatments within the mCSPC phase.

46 **Figure S6.** *Sequence of treatments from mCSPC diagnosis to death or end of follow-up,*
47 *2017–2023, IPÖ cohort*
48

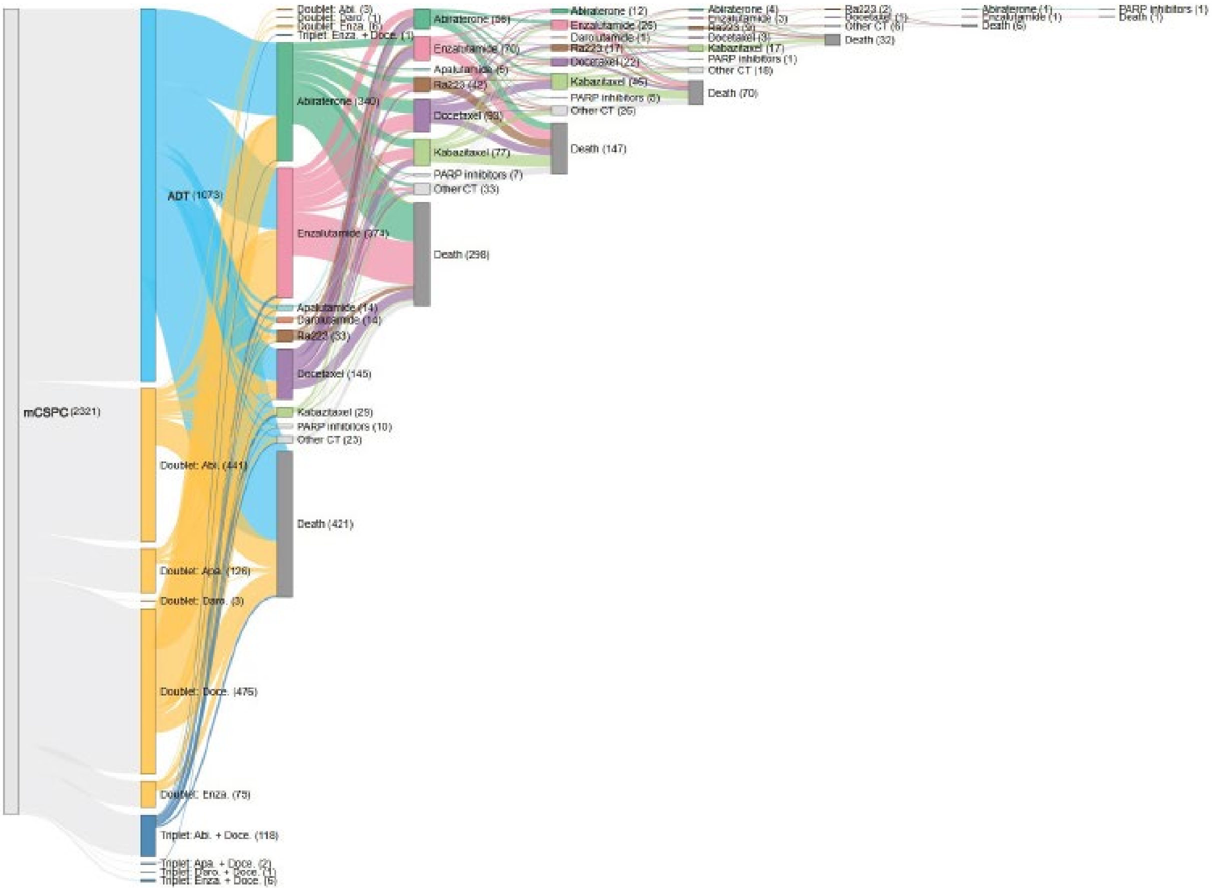

49

50 Abi, abiraterone; ADT, androgen-deprivation therapy; Apa, apalutamide; CT, chemotherapy;
51 Daro, darolutamide; Doce, docetaxel; Enza, enzalutamide; IPÖ, Individuell patientöversikt;
52 mCSPC, metastatic castration-sensitive prostate cancer; PARP, poly (ADP-ribose)
53 polymerase; Ra223, radium-223 dichloride.
54
